# Cumulative exposure to elevated blood pressure better predicts cardiovascular disease risk in rural Chinese adults

**DOI:** 10.3389/fpubh.2022.1006220

**Published:** 2022-10-04

**Authors:** Jiangbo Wang, Shiru Zhang, Yundi Jiao, Liqiang Zheng, Yingxian Sun, Zhaoqing Sun

**Affiliations:** ^1^Department of Cardiology, Shengjing Hospital of China Medical University, Shenyang, China; ^2^School of Public Health, Shanghai Jiao Tong University School of Medicine, Shanghai, China; ^3^Department of Cardiology, The First Affiliated Hospital of China Medical University, Shenyang, China

**Keywords:** hypertension, cardiovascular disease, stroke, myocardial infarction, blood pressure

## Abstract

**Background:**

Traditional risk estimations for cardiovascular disease (CVD) are based on current blood pressure (BP); however, whether cumulative exposure to elevated BP among rural individuals has additional prognostic value is unclear. We aimed to validate the association of cumulative BP with CVD occurrence and assess the prognostic value of cumulative BP in CVD risk prediction.

**Methods:**

A total of 13,057 participants who underwent three examinations from 2004 to 2010 were included in this rural epidemiological study and followed up until 2017. Cumulative BP was defined as the sum of the product of the average BP values between consecutive examinations and the time interval for each pair of successive tests prior to the follow-up period. CVD incidents that occurred during the follow-up period were noted and verified by qualified researchers. We used multivariate Cox models to assess the association of cumulative BP with CVD risk. The receiver operating characteristic curve was constructed to determine the predictive differentiation of single baseline BP measurements and cumulative BP values for CVD outcomes.

**Results:**

During the follow-up period, 1,312 participants underwent CVD incidents. We found that cumulative systolic BP (hazard ratio = 1.334, 95% confidence interval: 1.245, 1.430) and cumulative diastolic BP (hazard ratio = 1.253, 95% confidence interval: 1.168, 1.343) were associated with CVD incidence above and beyond that of the current BP. These stronger associations persisted for stroke, myocardial infarction, and CVD mortality. The area under the curve for the model increased significantly (*p* < 0.001) from 0.735 (0.720, 0.750) to 0.742 (0.728, 0.757) when integrating cumulative systolic BP instead of baseline systolic BP.

**Conclusion:**

Cumulative BP in Chinese rural adults showed a stronger association with CVD incidence than that of current BP. Furthermore, cumulative BP slightly improved the predictive performance for CVD. Our findings underline the incremental predictive value of cumulative BP in CVD risk assessment among Chinese rural adults.

## Introduction

Hypertension is one of the top five risk factors for disease burden worldwide, and approximately half of all people in China aged 35–75 years have hypertension (1, 2). In addition, hypertension remains the highest risk factor for cardiovascular disease (CVD) (3, 4). According to findings from the Blood Pressure Lowering Treatment Trialists' Collaboration study, each 5-mmHg decrease in systolic blood pressure (BP) reduces the risk of cardiovascular events by ~10% (5). Therefore, elevated BP poses a serious threat to public health.

However, the current frequently used prediction models for atherosclerotic CVD use baseline BP levels measured at a single time point, which do not consider the long-term fluctuations and cumulative exposure to BP levels (6–8). It is widely acknowledged that CVD occurrence arises from the progressive load of risk factors on vascular and cardiac tissues over time (9, 10). Although previous studies have discovered that cumulative BP, which incorporates both the intensity and duration of long-term BP recordings, has a more informative predictive value for CVD risk than baseline BP measurements in different populations, the generalization of this discovery remains somewhat limited because of the specific occupational population (11) and age of the study populations (12, 13). Whether cumulative BP has incremental predictive value at the time of CVD risk assessment among rural populations, the key target audience in primary care with higher morbidity and mortality than other populations in China (14, 15), remains uncertain.

Therefore, our main objective was to validate the effect of cumulative BP on subsequent CVD risk among 13,057 individuals in this prospective cohort study conducted in rural areas of China. Moreover, we assessed the predictive deviation of cumulative vs. baseline BP for the incidence of CVD during the follow-up period.

## Methods

### Study design and population

The present analysis was based on a previously reported large-scale epidemiological cohort study (16, 17). In brief, this study adopted a random stratified cluster-sampling scheme to investigate the prevalence, incidence, and natural history of cardiovascular risk factors, and 84 rural villages in eight towns from diverse geographic regions were identified according to the population. A total of 45,925 individuals aged ≥ 35 years were enrolled as a representative sample. The initial anthropometric data of the overall cohort were gathered between 2004 and 2006, and three additional follow-up examinations were completed in 2008, 2010, and 2017. Among the total population, 3,883 participants were excluded because they lacked contact information or refused to participate in follow-up. Ultimately, 42,042 participants were eligible to participate in the follow-up survey.

**Figure 1:**
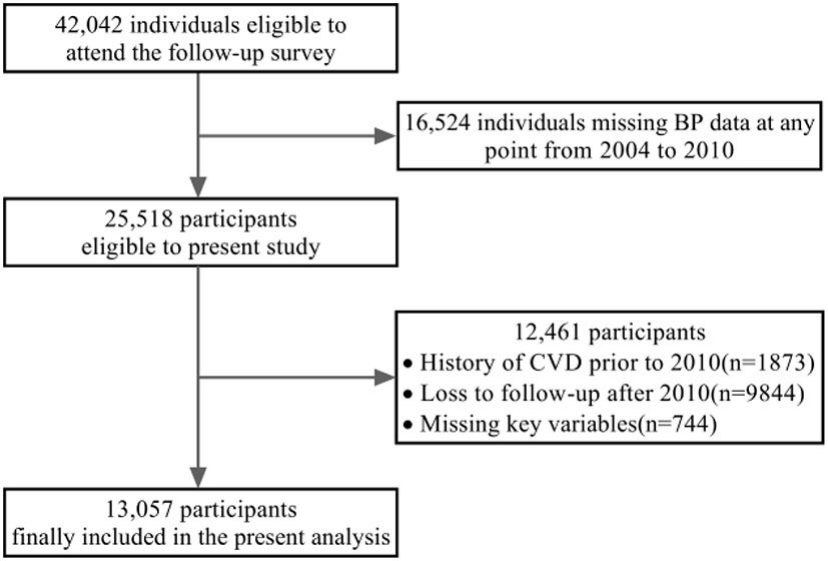
The flowchart for the study design and population.

Individuals with missing data on BP level at least one time point from 2004 to 2010 and a history of CVD prior to 2010, as well as participants who dropped out after 2010, were excluded from the current study (Figure 1). Consequently, 13,057 individuals were ultimately included in the analysis.

### BP assessment

During each visit, three BP readings were obtained and recorded by competent technicians using a calibrated electronic sphygmomanometer (HEM-741C; OMRON Corporation, Kyoto, Japan) after a minimum of 5 min of rest. The average of three BP measurements was used for further analysis. Participants were instructed to abstain from drinking alcohol, smoking cigarettes, drinking tea, and engaging in strenuous activities for at least 30 min prior to each examination. Cumulative BP, the major exposure variable, was represented in mmHg × years and defined for each participant as the sum of the product of the average BP values between consecutive examinations and the time interval for each pair of successive tests. Systolic and diastolic BP were calculated.

### Data collection

Qualified researchers performed rigorous measurements and administered standard questionnaires to gather demographic data (age, sex, ethnicity, and education level); lifestyle factors (smoking status, alcohol consumption, and sodium intake); medical history (stroke, coronary heart disease, diabetes mellitus, and hyperlipidaemia); and anti-hypertensive medication use for every participant. Based on a standardized measurement protocol, participants were instructed to wear light-weight clothing and bare feet for height and weight measurements, which were then used to calculate body mass index. Sodium intake was estimated by dividing the total household sodium consumption by the total number of family members (16). Current drinking was defined as consumption of more than 8 grams of alcohol per week, which was derived by converting the weekly beer, wine, and liquor intake (18). Current smoking was defined as smoking at least one cigarette per day in the previous year. Primary school or below, middle school, and high school or above were used to characterize education levels. In addition, throughout each examination, participants provided information on their previous history of diabetes, coronary heart disease, and stroke, as well as data on contemporaneous medication usage, verified by medical records.

### Outcomes assessment

The outcomes during the follow-up period were independently reviewed and confirmed *via* autopsy reports, death certificates, and medical records by the endpoint assessment committee in which members were blinded to the baseline risk factor information of the study participants. The primary study outcome was incident CVD, including non-fatal myocardial infarction (MI), non-fatal stroke, and CVD mortality. If more than one incident occurred in a participant during the follow-up period, the first occurrence was deemed the endpoint. In addition, MI, stroke, and CVD mortality functioned as independent outcomes. MI was diagnosed based on the consensus document of the Joint European Society of Cardiology/American College of Cardiology Committee by combining laboratory markers with electrocardiographic signs or clinical symptoms (19). According to the World Health Organization's Multinational Monitoring of Trends and Determinants in Cardiovascular Disease criteria, stroke is defined as a rapidly developing signs of a focal (or global) disturbance of cerebral function that lasts longer than 24 h without a clear non-vascular cause (unless interrupted by surgery or death). This definition covers individuals who exhibit clinical symptoms and signs indicative of a complete stroke, including ischaemic and haemorrhagic stroke (intra-cerebral and sub-arachnoid hemorrhage stroke). All stroke cases were diagnosed using computed tomography, magnetic resonance imaging (including diffusion images), brain magnetic resonance angiography, or carotid duplex imaging (20). The follow-up visits ended in December 2017.

### Statistical analysis

The demographic variables, lifestyle factors, medical history, anti-hypertensive medication use, and baseline BP readings were obtained from data collected during the examination in 2010, the beginning of the follow-up period in the present study. The overall characteristics are reported as the mean ± standard deviation for continuous variables and frequencies and proportions for categorical variables. The Kaplan-Meier method was used to calculate the cumulative incidence of CVD by tertiles of cumulative BP among participants; the long-rank test was performed for comparisons among tertiles. The association between baseline or cumulative BP and incident CVD was assessed using Cox proportional hazard models. Continuous baseline BP measurements and cumulative BP values scaled per standard deviation change were separately fitted to the model. The proportional hazards assumption in the Cox proportional hazards models was confirmed using Schoenfeld residuals. We constructed three models: unadjusted model A; adjusted model B with covariates of age, sex, body mass index, history of hyperlipidaemia and diabetes, anti-hypertensive medication use, smoking and drinking status, sodium intake, family history of hypertension, and education level; and model C that simultaneously included baseline BP data and cumulative BP measurements (either systolic or diastolic BP) after adjusting for all factors in model B. Individual predictive values determined by the receiver operating characteristic curve were used to assess the predictive value of baseline and cumulative BP for CVD incidence. The areas under the curves were computed to assess and compare the improvement in prediction ability when cumulative BP measurements obtained before follow-up were included in the adjusted model, rather than the BP readings obtained at the beginning of follow-up. In addition, models for the association of cumulative BP with incident CVD outcomes were implemented for the age sub-groups. All statistical analyses were performed using STATA version 16.0 (StataCorp LLC, College Station, TX, USA); *p*-values < 0.05 were considered statistically significant.

## Results

**Figure 2:**
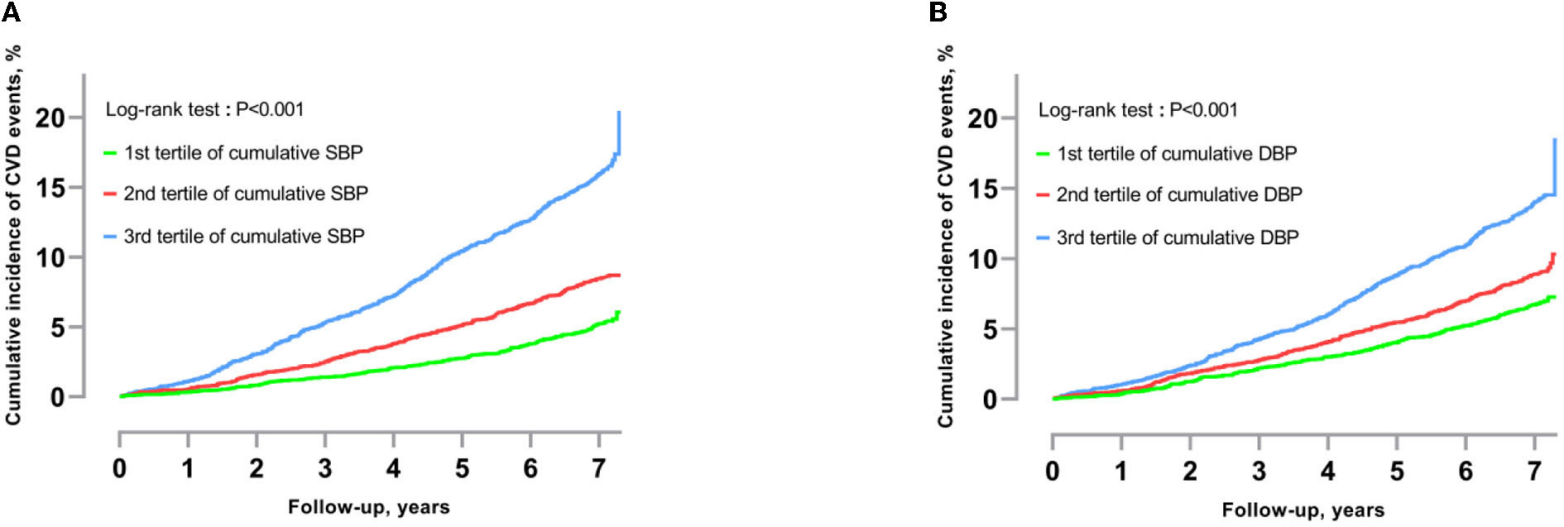
**(A)** Cumulative incidence of CVD by tertile of cumulative systolic BP. **(B)** Cumulative incidence of CVD by tertile of cumulative diastolic BP. The cumulative incidence of CVD by tertile of cumulative BP was calculated using the Kaplan–Meier method. The log-rank test was used to calculate the *p*-value (<0.001). SBP, Systolic blood pressure; DBP, diastolic blood pressure; CVD, cardiovascular disease.

The main participant characteristics across tertiles of cumulative systolic BP are shown in Table 1. Among the 13,057 study participants, the mean age was 52.1 ± 10.1 years and 51.5% (6,720) were men. At the onset of the follow-up period, the systolic and diastolic BP means were 130.5 and 80.8 mmHg, respectively. The mean cumulative BP measurements were 638.3 and 396.9 mmHg × years for systolic and diastolic BP, respectively. Individuals with higher cumulative systolic BP were more likely to be male, older, to smoke and drink alcohol, to have a higher body mass index, and to be less educated. The cumulative incidence curves of primary outcomes according to cumulative systolic and diastolic BP categories are shown in Figures 2A,B, respectively. Individuals with the highest cumulative BP had the highest incidence of CVD (*p* < 0.001).

**Table 1:** The characteristics of the study population by the tertile of cumulative systolic BP.

**Characteristics**	**Total (*n* = 13,057)**	**Tertile of cumulative systolic BP**			***P-value***
		**Tertile 1** **(*n* = 4,348)**	**Tertile 2 (*n* = 4,349)**	**Tertile 3** **(*n* = 4,360)**	
Age, (years)	52.1 ± 10.1	48.7 ± 8.7	51.4 ± 9.3	56.3 ± 10.8	<0.001
Men, *n* (%)	6,720 (51.5)	1,723 (39.6)	2,504 (57.6)	2,493 (57.2)	<0.001
**Ethnicity**, ***n*** **(%)**					<0.001
Han	10,061 (77.1)	3,498 (80.5)	3,413 (78.5)	3,150 (72.2)	
Mongolian	2,811 (21.5)	778(17.9)	879(20.2)	1,154 (26.5)	
Other	185 (1.4)	72 (1.7)	57 (1.3)	56 (1.3)	
Body mass index, kg/ m^2^	23.8 ± 3.1	23.4 ± 2.8	23.6 ± 2.6	24.3 ± 3.6	<0.001
Salt intake, g/d	15.2 ± 12.3	14.7 ± 12.8	14.6 ± 11.8	16.2 ± 12.2	<0.001
Current smoking, *n* (%)	4,763 (36.5)	1,331 (30.6)	1,747 (40.2)	1,685 (38.6)	<0.001
Current drinking, *n* (%)	3,993 (30.6)	1,057 (24.3)	1,496 (34.4)	1,440 (33.0)	<0.001
Antihypertensive drugs use, *n* (%)	841 (6.4)	25 (0.6)	105 (2.4)	711 (16.3)	<0.001
History of diabetes, *n* (%)	68 (0.5)	11 (0.3)	19 (0.4)	38 (0.9)	<0.001
Family history of hypertension, *n* (%)	1,538 (11.8)	399 (9.2)	500 (11.5)	639 (14.7)	<0.001
History of hyperlipidemia, *n* (%)	289 (2.2)	40 (0.9)	81 (1.9)	69 (3.9)	<0.001
**Education level**					<0.001
Primary school or below	5,211 (39.9)	1,477 (34.0)	1,658 (38.1)	2,076 (47.6)	
Middle school	7,143 (54.7)	2,660 (61.2)	2,448(56.3)	2,035 (46.7)	
High school or above	703 (5.4)	211 (4.9)	243 (5.6)	249 (5.7)	
**Blood pressure**					
Systolic BP, mmHg	130.5 ± 14.6	120.0 ± 9.6	129.3 ± 9.4	142.2 ± 14.5	<0.001
Diastolic BP, mmHg	80.8 ± 9.6	76.0 ± 8.0	79.9 ± 8.4	86.3 ± 9.5	<0.001
Cumulative systolic BP, mmHg*years	638.3 ± 57.1	581.2 ± 24.9	633.0 ± 12.4	700.7 ± 42.5	<0.001
Cumulative diastolic BP, mmHg*years	396.9 ± 36.5	372.0 ± 26.1	392.0 ± 25.7	426.6 ± 33.4	<0.001

Population characteristics are presented as mean ± standard deviation for continuous variables, and frequencies and proportions for categorical variables. *P*-value represents multiple-comparison tests for between-group differences. BP, blood pressure.

Over the course of follow-up, a total of 1,312 participants experienced CVD events: 954 strokes, 309 MIs, and 690 CVD deaths. Table 2 shows the association of baseline BP levels assessed at a single time point and cumulative BP data on a continuous scale with CVD events. BP and cumulative BP were both significantly associated with the incidence of CVD, stroke, MI, and CVD mortality in the unadjusted hazards models. Even after adjustments for confounding variables in the Cox models, the results indicated that baseline systolic BP [hazard ratio (HR) = 1.161, 95% confidence interval (CI): 1.097–1.229], baseline diastolic BP (HR = 1.063, 95% CI: 1.003–1.126), cumulative systolic BP (HR = 1.313, 95% CI: 1.242–1.387), and cumulative diastolic BP (HR = 1.200, 95% CI: 1.134–1.270) were significantly associated with CVD incidence. The association also remained statistically significant for the other endpoints. Nonetheless, once current and cumulative BP were incorporated into the model concurrently, a higher risk association was demonstrated for cumulative BP when we assessed separately for incident CVD, stroke, MI, and CVD mortality, whereas the association with baseline BP (systolic or diastolic) was not significant. As shown in Table 3, the association between cumulative BP and long-term CVD risk persisted even when the models were stratified by age sub-groups.

**Table 2:** Association of cumulative or baseline blood pressure levels (continuous scale) with hazards of cardiovascular disease (per 1 standard deviation).

	**CVD**	**Stroke**	**MI**	**CVD mortality**
	**Hazard ratio (95% confidence interval)**			
**Model A: Unadjusted**				
Systolic BP	1.419 (1.354, 1.487)	1.403 (1.327, 1.482)	1.450 (1.320, 1.593)	1.530 (1.440, 1.626)
Diastolic BP	1.244 (1.183, 1.308)	1.243 (1.172, 1.319)	1.240 (1.117, 1.376)	1.288 (1.203, 1.378)
Cumulative systolic BP	1.614 (1.546, 1.685)	1.581 (1.502, 1.664)	1.631 (1.494, 1.780)	1.794 (1.702, 1.892)
Cumulative diastolic BP	1.421 (1.351, 1.494)	1.401 (1.321, 1.486)	1.429 (1.289, 1.583)	1.496 (1.398, 1.600)
**Model B: Adjusted**				
Systolic BP	1.161 (1.097, 1.229)	1.178 (1.102, 1.260)	1.141 (1.017, 1.280)	1.181 (1.096, 1.273)
Diastolic BP	1.063 (1.003, 1.126)	1.075 (1.004, 1.150)	1.039 (0.921, 1.171)	1.072 (0.990, 1.160)
Cumulative systolic BP	1.313 (1.242, 1.387)	1.332 (1.248, 1.421)	1.239 (1.105, 1.389)	1.368 (1.272, 1.472)
Cumulative diastolic BP	1.200 (1.134, 1.270)	1.200 (1.122, 1.282)	1.179 (1.051, 1.323)	1.211 (1.122, 1.307)
**Model C: Adjusted, including baseline BP**				
Systolic BP	0.972 (0.905, 1.045)	0.980 (0.900, 1.067)	1.004 (0.868, 1.162)	0.966 (0.879, 1.062)
Diastolic BP	0.927 (0.863, 0.997)	0.943 (0.866, 1.027)	1.908 (0.782, 1.053)	0.929 (0.842, 1.025)
Cumulative systolic BP	1.334 (1.245, 1.430)	1.348 (1.242, 1.463)	1.236 (1.071, 1.426)	1.396 (1.275, 1.529)
Cumulative diastolic BP	1.253 (1.168, 1.343)	1.241 (1.143, 1.347)	1.246 (1.081, 1.436)	1.262 (1.150, 1.386)

Cox models assess the association of systolic BP, diastolic BP, cumulative systolic BP, and cumulative diastolic BP measures for incident CVD, stroke, MI, and CVD mortality. CI, confidence interval; MI, myocardial infarction; CVD, cardiovascular disease; BP, blood pressure.

Model A was unadjusted.

Model B was adjusted for age, gender, body Mass Index, history of hyperlipidemia, diabetes, antihypertensive medication usage, smoking, drinking, salt intake, family history of hypertension, and education level.

Model C was adjusted for age, gender, body Mass Index, history of hyperlipidemia, diabetes, antihypertensive medication usage, smoking, drinking, salt intake, family history of hypertension, education level and baseline BP (either systolic BP or diastolic BP).

**Table 3:** Association of cumulative systolic blood pressure levels (continuous scale) with hazards of cardiovascular diseases – subgroups analysis (per 1 standard deviation).

**Hazard ratio (95% CI) for incident CVD**	**Model A Unadjusted**	**Model B** **Adjusted**	**Model C Adjusted, including baseline BP**
**Age** ** <45 (*****n*** **=** **3,592)**			
Cumulative systolic BP	1.474 (1.247, 1.743)	1.423 (1.168, 1.734)	1.690 (1.309, 2.183)
Cumulative diastolic BP	1.431 (1.221, 1.678)	1.392 (1.161, 1.669)	1.532 (1.214, 1.933)
**Age 45–59 (*****n*** **=** **6,638)**			
Cumulative systolic BP	1.470 (1.356, 1.595)	1.394 (1.269, 1.531)	1.396 (1.239, 1.573)
Cumulative diastolic BP	1.363 (1.255, 1.479)	1.280 (1.169, 1.403)	1.352 (1.207, 1.514)
**Age** **>60 (*****n*** **=** **2,827)**			
Cumulative systolic BP	1.340 (1.258, 1.428)	1.246 (1.157, 1.342)	1.243 (1.134, 1.363)
Cumulative diastolic BP	1.167 (1.085, 1.255)	1.123 (1.038, 1.215)	1.153 (1.048, 1.269)

Cox models assess the association of cumulative systolic BP, and cumulative diastolic BP measures for incident CVD. CI, confidence interval; CVD, cardiovascular disease; BP, blood pressure.

Model A was unadjusted.

Model B was adjusted for age, gender, body Mass Index, history of hyperlipidemia, diabetes, antihypertensive medication usage, smoking, drinking, salt intake, family history of hypertension, and education level.

Model C was adjusted for age, gender, body Mass Index, history of hyperlipidemia, diabetes, antihypertensive medication usage, smoking, drinking, salt intake, family history of hypertension, education level and baseline BP (either systolic BP or diastolic BP).

**Figure 3:**
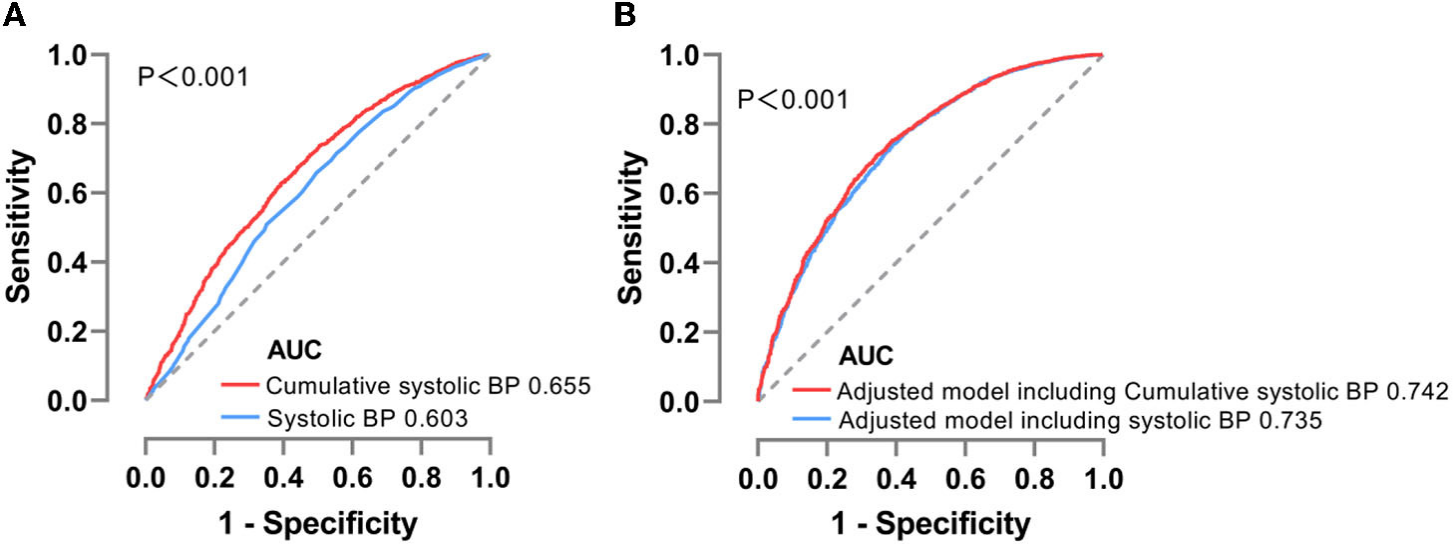
**(A)** ROC analysis for baseline or cumulative systolic BP distinguishing CVD incidence. **(B)** ROC analysis for the inclusion of baseline or cumulative systolic BP to adjusted models predicting CVD incidence. ROC, receiver operating characteristic; AUC, area under curve; CVD, cardiovascular disease; BP, blood pressure.

In addition, we discriminated the improvement in predictive performance for CVD occurrence by conducting a receiver operating characteristic curve analysis. In a direct comparison of the effectiveness of baseline and cumulative systolic BP on risk estimation, the areas under the curve were 0.603 (95% CI: 0.586–0.620) and 0.655 (95% CI: 0.639–0.671), respectively, with a significant difference (*p* < 0.001) (Figure 3A). Moreover, the analysis results indicated that integrating cumulative systolic BP instead of systolic BP at a single time point in the model increased the area under the curve from 0.735 (0.720, 0.750) to 0.742 (0.728, 0.757), which was statistically significant (*p* < 0.001) even though this result only represented a minor improvement (0.7%) in prediction ability (Figure 3B).

## Discussion

In this prospective cohort of Chinese rural adults, cumulative BP was associated with an increased risk of CVD. Cumulative BP showed a significantly greater risk association with CVD incidence than that of baseline BP, even after stratification by age. Substituting cumulative systolic BP for baseline systolic BP in the models significantly improved the prediction performance for CVD, although the improvements were modest in magnitude.

The cumulative exposure to BP incorporated with the duration and severity of the BP level may provide additional insight into disease risk assessment. Previous studies have emphasized the importance of long-term exposure to elevated BP in the development of CVD. A previous study from the Lifetime Risk Pooling Project found that 10-year cumulative systolic BP exposure was a risk factor that surpassed current systolic BP for CVD and all-cause death during a median follow-up period of 12.9 years (21). The Coronary Artery Risk Development in Young Adults study discovered that quantified cumulative exposure to BP over 16 years of follow-up was associated with subsequent risks of heart failure, coronary heart disease, stroke, and CVD (12). In the present analysis, our findings are consistent with those of prior investigations and cover a wider age range of participants. The Kailuan Study demonstrated a higher CVD risk associated with cumulative BP across a follow-up period of 3 years for cardiovascular and cerebrovascular events among the Chinese occupational population (22). Compared with this study, ours had a longer follow-up period with a relatively sufficient amount of time for the development of CVD. Additionally, in the sub-group analysis stratified by age, cumulative BP remained associated with CVD incidence even after adjustment for baseline BP. This finding is highly consistent with those in the age sub-groups in the Lifetime Risk Pooling Project (21).

Moreover, our study revealed that the improvement in predictive performance for CVD outcomes in the adjusted models was modest. The Lifetime Risk Pooling Project also found that using long-term measures of cumulative BP instead of single measurements at baseline can modestly improve the ability of CVD risk prediction models to correctly classify individuals in terms of their risk for CVD (23). The Kailuan Study demonstrated that the predictive value of baseline BP for the occurrence of cardiovascular events was only slightly lower than that of the cumulative BP (11). Considering that single baseline BP is more accessible, baseline BP may still be a useful marker for CVD risk assessment.

The rising prevalence of hypertension in low- and middle-income nations over the past few decades has increased the burden of primary prevention, directly resulting in an ever-increasing number of individuals with increased CVD risk (24, 25). Individualized CVD risk assessment in conjunction with BP levels has become the basis for determining anti-hypertensive treatment strategies (6, 8), and have been proven more effective and cost-effective (26, 27). Cumulative BP, which considers the intensity and duration of BP levels, has a higher predictive value for CVD risk than that of current BP. Individuals with similar BP levels may have different long-term risks for CVD owing to variations in their cumulative exposure to BP; therefore, cumulative BP may be used to stratify individuals based on their CVD risk. Since BP is measured frequently and is widely available in longitudinal electronic health record data, cumulative BP can be applied in primary health systems with adequate electronic data capabilities (23).

This study had several limitations. First, while there were 13,057 participants in the study sample, 26,368 were excluded because they were lost to follow-up at any time between 2004 and 2017, which may have resulted in bias. Second, cumulative BP only marginally improved the risk-prediction model. Consequently, further studies in different populations are needed to investigate the applicability of cumulative BP. Third, some incidents may not have been reported because of vague clinical signs. Therefore, the actual number of cases might have been underestimated in this study. Finally, this study was conducted on a rural population in northeastern China, and whether the findings can be generalized to other populations needs to be verified.

## Conclusion

Cumulative BP in Chinese rural adults showed a stronger association with CVD incidence than that of current BP. Furthermore, cumulative BP slightly improved the predictive performance for CVD. Our findings underline the incremental predictive value of cumulative BP in CVD risk assessment among Chinese rural adults.

## Data availability statement

The raw data supporting the conclusions of this article will be made available by the authors, without undue reservation.

## Ethics statement

The studies involving human participants were reviewed and approved by the Research Ethics Committee of China Medical University. The patients/participants provided their written informed consent to participate in this study.

## Author contributions

JW wrote the manuscript. ZS and YS conceived and designed the study. SZ and LZ supervised the statistical analysis. YJ collected all relevant data and contributed to the discussion. All authors have read and approved the final version of the manuscript.

## Funding

This study was supported by the National Key R&D Program of China (Grant #2018YFC1311600), Liaoning Revitalization Talents Program (Grant #XLYC1808036), and 345 Talent Project of Shengjing Hospital.

## Conflict of interest

The authors declare that the research was conducted in the absence of any commercial or financial relationships that could be construed as a potential conflict of interest.

## Publisher's note

All claims expressed in this article are solely those of the authors and do not necessarily represent those of their affiliated organizations, or those of the publisher, the editors and the reviewers. Any product that may be evaluated in this article, or claim that may be made by its manufacturer, is not guaranteed or endorsed by the publisher.
